# Colchicine-Tolerant vs. Resistant Familial Mediterranean Fever: Comparative Analysis of Clinical, Psychosocial Characteristics and Quality of Life

**DOI:** 10.3390/jcm15051784

**Published:** 2026-02-27

**Authors:** Zeynep Kaya, Sinem Sag, Mehmet Nur Kaya, Sezgin Zontul, Servet Yolbas

**Affiliations:** 1 Division of Rheumatology, Department of Internal Medicine, Istanbul Training and Research Hospital, Istanbul 34098, Turkey; 2 Division of Rheumatology, Department of Physical Medicine and Rehabilitation, Istanbul Fatih Sultan Mehmet Training and Research Hospital, Istanbul 34758, Turkey; drsinemyamac@yahoo.com; 3 Division of Rheumatology, Department of Internal Medicine, Faculty of Medicine, Balikesir University, Balikesir 10010, Turkey; mehmetnurkaya@yahoo.com; 4 Division of Rheumatology, Department of Physical Medicine and Rehabilitation, Inonu University Faculty of Medicine, Malatya 44280, Turkey; sezgin.zontul@inonu.edu.tr; 5 Division of Rheumatology, Department of Internal Medicine, Inonu University Faculty of Medicine, Malatya 44280, Turkey; servet.yolbas@inonu.edu.tr

**Keywords:** familial mediterranean fever, colchicine resistance, disease activity, quality of life, psychological burden

## Abstract

**Background/Objectives**: Familial Mediterranean Fever (FMF) is a chronic autoinflammatory disease in which some patients develop resistance to colchicine, resulting in persistent attacks and increased disease burden. This study aimed to compare clinical characteristics, disease activity, psychological status, and quality of life between colchicine-tolerant and colchicine-resistant FMF patients, and to identify clinical factors independently associated with colchicine resistance. **Methods**: This exploratory cross-sectional observational study was conducted in 120 FMF patients followed at a tertiary rheumatology center. Patients were classified as colchicine-tolerant or colchicine-resistant. Disease activity and damage were assessed using the International Severity Scoring System for FMF (ISSF) and the Autoinflammatory Disease Damage Index (ADDI). Quality of life was evaluated using the FMF–Health-Related Quality of Life (FMF-HQL) and WHO Quality of Life–BREF (WHOQoL-BREF) questionnaires. Anxiety and depression were assessed using the Hospital Anxiety and Depression Scale (HADS). **Results**: Colchicine-resistant patients had significantly higher attack frequency and disease activity scores (*p* < 0.001). Quality of life was impaired, with higher FMF-HQL and lower WHOQoL-BREF scores across all domains (*p* < 0.001). Anxiety and depression scores were also higher. ISSF and Doctor Global Assessment (DGA) were independently associated with colchicine resistance. **Conclusions**: Colchicine resistance in FMF was associated with increased disease activity, impaired quality of life, and greater psychological burden.

## 1. Introduction

Familial Mediterranean Fever (FMF) is a hereditary autoinflammatory disorder characterized by recurrent febrile attacks and serosal inflammation (e.g., peritonitis, pleuritis, arthritis) that primarily affects populations of Eastern Mediterranean descent [1].

The disease is caused by mutations in the *MEFV* gene, which encodes pyrin, a key regulator of innate immune responses and inflammasome activation [2]. Dysregulated interleukin-1 (IL-1) signaling resulting from pyrin dysfunction leads to exaggerated inflammatory responses and recurrent inflammatory attacks [3].

Colchicine remains the mainstay of FMF therapy, effectively reducing the frequency and severity of attacks, suppressing subclinical inflammation, and preventing long-term complications such as AA amyloidosis [4]. Despite its efficacy, approximately 5–10% of FMF patients exhibit poor or insufficient responses, often labeled as *colchicine resistance* [5]. Colchicine resistance is associated with increased morbidity, higher risk of complications, and the need for alternative therapeutic strategies, including IL-1–targeted biologic agents [6,7].

Previous studies have shown that colchicine-resistant FMF is not fully explained by demographic or genetic factors alone, suggesting that disease activity, cumulative damage, and patient-reported outcomes may play a central role in defining this subgroup [8,9]. Beyond physical symptoms, FMF has been shown to significantly impair health-related quality of life and psychosocial well-being, particularly in patients with frequent attacks and uncontrolled disease [10,11]. Anxiety and depression are increasingly recognized as common comorbidities in FMF and are closely linked to disease activity and functional impairment [12,13].

Although the negative impact of FMF on quality of life is well established, data specifically addressing the combined effects of colchicine resistance, disease activity, psychological burden, and multidimensional QoL measures remain limited. Therefore, this study aimed to compare colchicine-tolerant and colchicine-resistant FMF patients in terms of demographic, clinical, laboratory, and psychosocial characteristics, including quality of life, anxiety, depression, and work-related impairment. Additionally, we sought to identify clinical factors independently associated with colchicine resistance using validated clinical activity and impairment scores, with the goal of supporting early recognition and personalized management strategies.

## 2. Materials and Methods

### 2.1. Study Design and Participants

This exploratory cross-sectional observational study was conducted involving 120 adult patients diagnosed with FMF according to the Tel-Hashomer criteria and consecutively admitted to our hospital between 2025 and 2026. All data collection was completed prior to manuscript submission [14].

Patients were stratified into two groups according to colchicine response: colchicine-tolerant and colchicine-resistant. Colchicine resistance was defined according to modified Delphi consensus criteria as the occurrence of ≥1 attack per month over a 3-month period despite adherence to an adequate dose of colchicine, or persistent laboratory inflammation between attacks [1].

Inclusion criteria were: confirmed diagnosis of FMF, age ≥ 18, stable colchicine treatment for at least 6 months. Exclusion criteria included: concurrent autoimmune or autoinflammatory diseases, incomplete medical records, nonadherence to colchicine therapy.

Demographic and clinical data including age, sex, body mass index, age at symptom onset, age at diagnosis, family history, clinical manifestations during attacks, colchicine dose and duration, and attack frequency over the last 1, 3, and 12 months were recorded. Genetic data, including *MEFV* mutation status, were retrieved from patients’ medical records. Mutation types, including heterozygous and homozygous variants, were documented, with particular attention to exon 10 mutations such as M694V, M680I, and V726A. Patients were classified as colchicine-tolerant or colchicine-resistant. Laboratory parameters including erythrocyte sedimentation rate, C-reactive protein, serum amyloid A, complete blood count indices, and 24 h proteinuria were obtained using standard laboratory methods. Disease activity and severity were assessed using multiple validated instruments: the International Severity Scoring System for FMF (ISSF), a composite score evaluating attack frequency, clinical manifestations, and disease complications; the Autoinflammatory Disease Damage Index (ADDI), which measures cumulative and irreversible organ damage related to autoinflammatory disease; the Visual Analog Scale (VAS), used to quantify patient-reported disease activity on a 0–10 scale. Disease activity was additionally evaluated using the Patient Global Assessment (PGA) and Doctor Global Assessment (DGA), which are widely used global measures of overall disease activity. PGA reflects the patient’s subjective perception of their overall disease activity at the time of evaluation. Patients were asked to rate their overall disease activity using a 10 cm VAS, ranging from 0 (no disease activity) to 10 (maximum disease activity imaginable), considering their symptoms, functional status, and general well-being. DGA represents the treating physician’s overall evaluation of disease activity based on clinical examination, patient-reported symptoms, attack frequency, physical findings, and laboratory parameters. The physician rated disease activity using a 10 cm VAS, ranging from 0 (no disease activity) to 10 (maximum disease activity), reflecting the physician’s integrated clinical judgment of current disease severity.

Health-related quality of life was evaluated using the Familial Mediterranean Fever–Health-Related Quality of Life (FMF-HQL) questionnaire, a disease-specific instrument assessing physical, emotional, and social impact of FMF, with higher scores indicating worse quality of life, and the World Health Organization Quality of Life–BREF (WHOQoL-BREF), which assesses physical, psychological, social, and environmental domains of general quality of life. Psychological status was assessed using the Hospital Anxiety and Depression Scale (HADS), consisting of anxiety (HADS-A) and depression (HADS-D) subscales, each ranging from 0 to 21, with higher scores indicating greater symptom severity.

During the preparation of this manuscript, the authors utilized ChatGPT (GPT-5, OpenAI (San Francisco, CA, USA), 2025) for English language editing and formatting, in accordance with the journal guidelines. The authors have thoroughly reviewed and revised the output and take full responsibility for the content of this publication.

The study protocol was approved by the Istanbul Training and Research Hospital Ethics Committee (approval date: 5 December 2025, approval no: 293). All patients provided written informed consent prior to inclusion in the study. The study was carried out in full compliance with the ethical standards outlined in the Declaration of Helsinki.

### 2.2. Statistical Analysis

The statistical analyses were conducted using IBM SPSS Statistics for Windows, version 28 (IBM Corp., Armonk, NY, USA). The Kolmogorov–Smirnov test was used to evaluate whether the variables were normally distributed. Normally distributed variables were expressed as mean ± standard deviation (SD), skewed variables as median (interquartile range-IQR), and categorical variables as number and percentage (%). Patients’ demographic and clinical characteristics were compared between these two groups using the chi-square test or Fisher’s exact test (when chi-square test assumptions do not hold) for categorical data, the Mann–Whitney U test for non-normally distributed continuous data, and independent t-test for normally distributed continuous data. A forward stepwise multivariate regression model was developed, using factors that had significant associations with colchicine resistance in the univariate analysis. Statistical significance was defined as *p* < 0.05.

This study was designed as an exploratory observational study including all eligible patients during the study period, and a formal a priori sample size calculation was not performed.

## 3. Results

A total cohort of 120 patients with Familial Mediterranean Fever was evaluated and stratified into colchicine-tolerant and colchicine-resistant groups. Comparison of the baseline demographic characteristics revealed no statistically significant differences between the groups regarding age, sex distribution, age at symptom onset, age at diagnosis, body mass index, or family history (all *p* > 0.05) (Table 1). Likewise, there was no significant difference in MEFV mutation frequencies, with heterozygous M694V being the most common variant in both groups. Assessment of clinical features during attacks demonstrated that fever was significantly more prevalent in colchicine-resistant patients (91.7% vs. 51.4%, *p* < 0.001). Additionally, the rates of arthralgia/arthritis (95.8% vs. 79.2%, *p* = 0.010) and nausea/vomiting (85.4% vs. 65.3%, *p* = 0.015) were notably higher in the resistant group. Other findings including peritonitis, pleuritis, erysipelas-like erythema, and myalgia showed no group differences (all *p* > 0.05). Laboratory characteristics, including inflammatory markers (ESR, CRP, SAA), hematologic indices (WBC, PLT, NEU, LYM), and renal involvement assessed by proteinuria were comparable between the two groups (all *p* > 0.05). However, colchicine-resistant patients required a significantly higher daily dose of colchicine than tolerant patients (1.7 ± 0.3 mg vs. 1.5 ± 0.3 mg; *p* = 0.012).

Disease activity and attack burden markers of FMF disease activity, including ISSF, ADDI, VAS, PGA, and DGA scores, were significantly elevated among colchicine-resistant individuals (all *p* < 0.001), indicating a higher burden of persistent disease (Table 2). Although median ISSF scores appeared similar, the distribution was significantly shifted toward higher severity in colchicine-resistant patients. Consistently, the number of attacks documented in the last 1 month, last 3 months, and the last 12 months were significantly higher in the resistant group (all *p* < 0.001). Colchicine-resistant patients exhibited significantly poorer quality of life outcomes, as reflected by higher FMF-HQL scores and significantly lower scores across all WHOQoL-BREF domains, including physical, psychological, social, and environmental quality of life (all *p* < 0.001). Psychological assessments further revealed that anxiety (HADS-A; *p* = 0.003) and depression (HADS-D; *p* = 0.001) scores were significantly higher among resistant patients, reflecting a more pronounced psychosocial burden.

Regression Analysis Univariate logistic regression identified multiple variables associated with colchicine resistance, including increased ISSF, ADDI, VAS, PGA, DGA, activity impairment, FMF-HQL scores, and WHOQoL domains (all *p* < 0.01). However, in the multivariate model, only ISSF (OR: 2.28, 95% CI: 1.19–4.38; *p* = 0.013) and DGA (OR: 1.70, 95% CI: 1.26–2.29; *p* < 0.001) remained independently associated with colchicine resistance (Table 3).

## 4. Discussion

In this study, we compared clinical characteristics, disease activity, psychological burden, and quality of life in colchicine-tolerant and colchicine-resistant patients with FMF. The main findings indicate that colchicine resistance is associated with a higher frequency of attacks, increased disease activity scores, impaired quality of life, and a greater burden of anxiety and depression; however, due to the cross-sectional design, causal relationships cannot be established, and these associations may be bidirectional.

Baseline demographic characteristics, including age, sex distribution, body mass index, age at disease onset, and age at diagnosis, were similar between the two groups. In addition, MEFV mutation profiles did not differ significantly, with M694V variants being the most common in both groups. These findings are consistent with previous studies demonstrating that colchicine resistance cannot be explained solely by demographic or genetic differences and may instead reflect persistent inflammatory activity and inadequate disease control [15,16]. Although we did not observe a significant difference in overall mutation distribution between colchicine-tolerant and colchicine-resistant patients, genetic factors may still contribute to disease severity and treatment response. Previous studies have suggested that certain high-risk genotypes, particularly M694V homozygosity and increased allelic burden, are associated with more severe disease phenotypes and a higher likelihood of colchicine resistance [17]. The lack of significant genetic differences in our cohort may reflect the complex and multifactorial nature of colchicine resistance, which likely involves interactions between genetic susceptibility, inflammatory pathways, and clinical and psychosocial factors. Additionally, our study evaluated mutation distribution descriptively and did not specifically analyze genotype severity or allelic burden. Future studies incorporating detailed genetic stratification may help clarify the role of genetic severity in predicting colchicine resistance.

Colchicine-resistant patients exhibited significantly higher rates of fever, arthralgia/arthritis, and gastrointestinal symptoms during attacks, as well as a markedly increased number of attacks in the preceding 1, 3, and 12 months. This higher attack burden was accompanied by significantly elevated disease activity indices, including ISSF, ADDI, VAS, PGA, and DGA scores. Similar associations between attack frequency, disease severity, and adverse clinical outcomes have been reported in both adult and pediatric FMF cohorts [18,19,20]. Importantly, multivariate regression analysis in our study identified ISSF and DGA as independently associated with colchicine resistance, reflecting their role as composite clinical indicators of disease severity and physician-assessed disease burden rather than causal determinants. These global assessment tools integrate multiple dimensions of disease burden beyond attack frequency alone, providing a more comprehensive assessment of overall disease severity. These findings further support the clinical utility of global disease activity assessments in identifying patients with a higher disease burden who may require closer monitoring and alternative therapeutic strategies.

A major finding of our study is the pronounced impairment in quality of life observed among colchicine-resistant patients; however, the cross-sectional design does not allow for the determination of whether reduced quality of life is a cause or consequence of increased disease activity.

FMF-HQL scores were significantly worse in the resistant group, and all WHOQoL domains—physical, psychological, social, and environmental—were markedly reduced. These results are in line with previous studies showing that FMF negatively affects health-related quality of life, particularly in patients with active disease and frequent attacks [11,21,22,23]. Large cohort studies using SF-36 and other validated instruments have demonstrated that pain, physical limitation, and social functioning are major determinants of reduced quality of life in FMF [10,11,24]. Our findings extend this literature by showing that colchicine resistance represents a subgroup with a substantially greater and more global quality of life impairment.

Psychological distress was significantly more prevalent in colchicine-resistant patients, as reflected by higher anxiety and depression scores. This observation is consistent with previous studies reporting increased rates of anxiety and depression among FMF patients compared with healthy controls [4,13,25,26]. Previous research has also demonstrated close associations between mood disorders, disease activity, and impaired quality of life in FMF [13,26]. Chronic inflammation, recurrent painful attacks, and uncertainty regarding disease control may contribute to the increased psychological burden observed in colchicine-resistant patients.

Despite modest absolute differences, these findings may still reflect clinically meaningful psychological burden in the context of chronic disease. Even mild-to-moderate elevations in anxiety and depression scores have been associated with reduced quality of life, impaired coping, and worse illness perception in chronic inflammatory diseases. These findings underscore the importance of incorporating psychological assessment into the comprehensive clinical management of patients with FMF.

Although inflammatory markers such as ESR, CRP, and serum amyloid A during the attack-free period did not differ significantly between the groups, quality of life and psychological measures were substantially worse in colchicine-resistant patients. This finding is consistent with prior studies suggesting that patient-reported outcomes and psychosocial well-being may be more sensitive indicators of disease burden than laboratory markers alone [11,23,27]. It also underscores the importance of incorporating patient-reported outcome measures into routine clinical assessment.

This apparent discrepancy between clinical disease burden and laboratory inflammatory markers suggests that colchicine resistance may manifest predominantly as increased clinical disease activity rather than persistent biochemical inflammation detectable during attack-free periods. Additionally, conventional inflammatory markers such as ESR, CRP, and SAA may not fully capture low-grade or subclinical inflammation. Furthermore, psychological factors such as anxiety, depression, and illness perception may influence symptom reporting and perceived disease severity, thereby contributing to the overall disease burden. These findings highlight the importance of comprehensive clinical assessment, including physician global assessment and patient-reported outcomes, in addition to laboratory parameters when evaluating disease activity and treatment response in patients with FMF.

Previous studies have shown that interleukin-1 antagonists improve quality of life, work productivity, and psychosocial functioning in colchicine-resistant FMF [28,29,30]. Although treatment outcomes were not directly evaluated in our study, the pronounced impairment observed in colchicine-resistant patients supports early recognition of inadequate colchicine response and timely consideration of biologic therapy.

Several limitations should be acknowledged. First, the cross-sectional design precludes causal inference between disease activity, colchicine resistance, psychological distress, and quality-of-life impairment. The observed associations may be bidirectional. For example, psychological distress such as anxiety and depression may influence symptom perception, disease reporting, and perceived disease severity, while persistent disease activity may also contribute to psychological burden and reduced quality of life. Therefore, these findings should be interpreted as associations rather than causal relationships. Longitudinal studies are needed to clarify the temporal and causal relationships between disease activity, psychological factors, and colchicine resistance. In addition, we did not specifically evaluate the proportion of patients exceeding established clinical cut-off values for anxiety and depression or calculate effect sizes, which may further clarify the clinical magnitude of psychological impairment.

Second, laboratory parameters were assessed at a single time point and may not fully reflect ongoing subclinical inflammation.

Third, a formal a priori power calculation was not performed, and the sample size was determined by the number of eligible patients during the study period. Given the multiple comparisons performed, there is a potential risk of type I error. Therefore, the findings should be interpreted within the context of an exploratory study, and larger prospective studies are needed to confirm these results.

Fourth, colchicine resistance is partially defined by ongoing disease activity and attack frequency, which may create conceptual overlap with disease activity indices such as ISSF, PGA, and DGA. Therefore, these measures should not be interpreted as causal predictors of colchicine resistance, but rather as independent clinical correlates reflecting the overall severity and physician-assessed burden of disease. The regression findings should be interpreted within the context of this definitional overlap and the cross-sectional design of the study. Future longitudinal studies incorporating genetic factors, biomarkers of subclinical inflammation, and psychosocial determinants may help identify mechanistically independent predictors of colchicine resistance.

Despite these limitations, the use of validated disease activity indices and quality of life instruments strengthens the clinical relevance of our findings.

## 5. Conclusions

Colchicine-resistant patients with Familial Mediterranean Fever exhibit a substantially higher disease burden than colchicine-tolerant individuals, despite comparable demographic characteristics, genetic mutation profiles, and laboratory inflammatory markers. Colchicine resistance is associated with increased attack frequency, more prominent musculoskeletal and gastrointestinal manifestations, and significantly higher disease activity and damage scores. In parallel, these patients experience marked impairment in both disease-specific and generic quality of life, accompanied by elevated levels of anxiety and depressive symptoms, highlighting the multidimensional impact of persistent disease activity beyond inflammatory parameters alone. Multivariate analysis identified the ISSF and DGA as independent clinical factors associated with colchicine resistance, reflecting the overall physician-assessed and composite burden of disease activity rather than causal predictors. Overall, these findings suggest that colchicine resistance in FMF is associated with increased disease activity, impaired quality of life, and greater psychological burden; however, due to the cross-sectional design, these findings reflect associations and do not establish causal relationships.

## Figures and Tables

**Table 1:** Demographic characteristics, clinical findings and genetic mutations of the patients.

Characteristics	Colchicine-Tolerant	Colchicine-Resistant	*p*
Male, *n* (%)	22 (30.6)	12 (25)	0.508
Age, years, mean (SD)	40.7 (12.2)	36.2 (13.2)	0.604
Age of symptom onset, years, mean (SD)	16.2 (11.2)	17 (12.9)	0.552
Age of diagnosis, years, mean (SD)	25.6 (12.6)	28.2 (14.1)	0.296
BMI, median (IQR)	26 (4.6)	24.9 (5.2)	0.263
Family history, *n* (%)	50 (69.4)	36 (75)	0.508
MEFV mutations, *n* (%)			
M694V/M694V	11 (15.3)	10 (20.8)	0.433
M694V heterozygous	21 (29.2)	15 (31.3)	0.807
V726A heterozygous	4 (5.6)	3 (6.3)	0.874
M680I/M680I	3 (4.2)	2 (4.2)	1.000
M680I heterozygous	7 (9.7)	4 (8.3)	0.796
E148Q heterozygous	4 (5.6)	3 (6.3)	0.874
M694V/M680I	9 (12.5)	4 (7.5)	0.472
M694V/E148Q	4 (5.6)	2 (4.2)	0.732
Others	6 (8.3)	1 (2.1)	0.152
No mutation	3 (4.2)	4 (8.3)	0.340
Fever	37 (51.4)	44 (91.7)	<0.001
Peritonitis	63 (87.5)	43 (89.6)	0.728
Arthralgia/arthritis	57 (79.2)	46 (95.8)	0.010
Myalgia	58 (80.6)	42 (87.5)	0.317
Nausea/vomiting	47 (65.3)	41 (85.4)	0.015
Erysipelas-like erythema	11 (15.3)	5 (10.4)	0.443
ESR (mm/h), median (IQR)	9 (12)	10 (14)	0.666
CRP (mg/L), median (IQR)	1.1 (2.6)	2 (3)	0.151
Serum amyloid A, median (IQR)	0.5 (0.5)	0.4 (0.4)	0.333
Proteinuria (mg/day), median (IQR)	90 (61)	86.5 (66)	0.885
HCT, median (IQR)	40.3 (5.5)	40.8 (4.8)	0.991
WBC, median (IQR)	6400 (2775)	6755 (2775)	0.120
PLT, median (IQR)	240 (89.7)	239 (104.3)	0.789
LYM, median (IQR)	2207 (1100)	2200 (915)	0.415
NEU, median (IQR)	4507 (1875)	4100 (1475)	0.207
Colchicine duration, years, median (IQR)	11 (8)	9.5 (11)	0.153
Colchicine dose (mg/day), mean (SD)	1.5 (0.3)	1.7 (0.3)	0.012
Number of attacks, median (IQR)			
In the last 1 month	0 (1)	1 (1)	<0.001
In the last 3 months	0 (1)	1 (2)	<0.001
In the last 12 months	2 (4)	4 (4)	<0.001
Pleuritis	42 (58.3)	27 (57.4)	0.924

SD, standard deviation; IQR, Interquartile Range.

**Table 2:** Disease activity and attack burden markers in patients with FMF.

Characteristics	Colchicine-Tolerant	Colchicine-Resistant	*p*
ISSF, median (IQR)	2 (0)	2 (1)	<0.001
ADDI, median (IQR)	1 (1)	2 (1)	<0.001
VAS, median (IQR)	3 (3)	4 (3)	<0.001
PGA, median (IQR)	3 (3)	4 (2)	<0.001
DGA, median (IQR)	2 (3)	3 (2)	<0.001
FMF-HQL, median (IQR)	24 (10)	32 (14)	<0.001
Activity imp, median (IQR)	20 (30)	40 (28)	<0.001
WHOQoL—Physical, median (IQR)	75 (23)	56 (31)	<0.001
WHOQoL—Psychological, median (IQR)	75 (23)	56 (21)	<0.001
WHOQoL—Social, median (IQR)	72 (20)	50 (24)	<0.001
WHOQoL—Environmental, median (IQR)	75 (12)	56 (19)	<0.001
HADS-A, median (IQR)	7 (4)	8.5 (3)	0.003
HADS-D, median (IQR)	5.5 (4)	7 (3)	0.001

**Table 3:** Univariate and multivariate logistic regression analysis of factors associated with colchicine resistance.

Covariate	Univariate			Multivariate		
	OR	*p*	95% CI (Lower-Upper)	OR	*p*	95% CI (Lower-Upper)
ISSF	3.32	<0.001	1.81–6.10	2.28	0.013	1.19–4.38
ADDI	2.22	<0.001	1.36–3.61			
VAS	1.51	<0.001	1.21–1.88			
PGA	1.58	<0.001	1.26–1.99			
DGA	1.92	<0.001	1.44–2.55	1.70	<0.001	1.26–2.29
FMF-HQL	1.09	<0.001	1.05–1.13			
Activity imp	1.05	<0.001	1.02–1.07			
WHOQoL—Physical	0.95	<0.001	0.93–0.98			
WHOQoL—Psychological	0.95	<0.001	0.92–0.97			
WHOQoL—Social	0.94	<0.001	0.94–0.97			
WHOQoL—Environmental	0.96	0.001	0.93–0.98			
HADS-A	1.25	0.003	1.25–1.45			
HADS-D	1.27	0.002	1.27–1.47			

## Data Availability

The data presented in this study are available on request from the corresponding author. The data are not publicly available due to privacy and ethical restrictions.

## Abbreviations

The following abbreviations are used in this manuscript:

AA	Amyloid A
ADDI	Autoinflammatory Disease Damage Index
BMI	Body Mass Index
CRP	C-reactive Protein
DGA	Doctor Global Assessment
ESR	Erythrocyte Sedimentation Rate
FMF	Familial Mediterranean Fever
FMF-HQL	Familial Mediterranean Fever–Health-Related Quality of Life
HADS	Hospital Anxiety and Depression Scale
HADS-A	Hospital Anxiety and Depression Scale–Anxiety
HADS-D	Hospital Anxiety and Depression Scale–Depression
HCT	Hematocrit
IL-1	Interleukin-1
ISSF	International Severity Scoring System for Familial Mediterranean Fever
IQR	Interquartile Range
LYM	Lymphocyte Count
MEFV	Mediterranean Fever Gene
NEU	Neutrophil Count
OR	Odds Ratio
PGA	Patient Global Assessment
PLT	Platelet Count
QoL	Quality of Life
SAA	Serum Amyloid A
SD	Standard Deviation
VAS	Visual Analog Scale
WBC	White Blood Cell Count
WHOQoL-BREF	World Health Organization Quality of Life–BREF
